# Performance-Based Assessment of Pakistani Regional Aggregates for Flexible Pavements Using Macro- and Micro-Characterization

**DOI:** 10.3390/ma19122535

**Published:** 2026-06-11

**Authors:** Fazli Karim, Nasir Khan, Md Arifuzzaman, Muhammad Imran Khan

**Affiliations:** 1 Department of Civil Engineering, Sarhad University of Science & Information Technology, Peshawar 25200, Pakistan; engr_fazli@yahoo.com; 2 Department of Civil & Environmental Engineering, Universiti Teknologi PETRONAS (UTP), Seri Iskandar 32610, Malaysia; nasir_22012207@utp.edu.my; 3 Department of Civil and Environmental Engineering, College of Engineering, King Faisal University, Al Ahsa 36362, Saudi Arabia; 4 Department of Civil Engineering, College of Engineering, Imam Mohammad Ibn Saud Islamic University (IMSIU), Riyadh 11564, Saudi Arabia

**Keywords:** aggregates ranking, geological sources, macro-level tests, micro-level tests, rutting

## Abstract

Aggregates comprise up to 95% of flexible pavement composition, critically influencing performance based on geological source and processing methods. In Pakistan, where approximately 264,175 km of roads carry 96% of inland freight, premium Margalla aggregates face increasing demand and depleting reserves, necessitating sustainable alternatives. This study comprehensively evaluates aggregates from five key quarries (Margalla, Malakand, Kohat, Swabi, and Besai) for highway suitability. Rigorous laboratory testing encompassed macro-level physical and mechanical properties and micro-characterization using Scanning Electron Microscopy (SEM), Energy Dispersive Spectroscopy (EDS), and Fourier Transform Infrared Spectroscopy (FTIR), alongside performance tests including Indirect Tensile Strength (ITS), rutting resistance, and fatigue analysis. Overall, Margalla aggregates exhibited the best performance, showing the lowest abrasion value (21%), highest Tensile Strength ratio (TSR) (82%), highest conditioned ITS (433.7 kPa), highest dynamic modulus (2120 MPa at 25 Hz), and the lowest rut depth (7.8 mm at 10,000 cycles). These superior properties are attributed to their favorable physical characteristics and high calcium content. Malakand and Kohat aggregates also demonstrated satisfactory performance, with TSR values of 79% and 76%, conditioned ITS values of 408.7 and 377.7 kPa, and rut depths of approximately 8.8 and 10.5 mm, respectively, indicating their suitability for medium-traffic pavements. In contrast, Swabi and Besai aggregates exhibited lower moisture resistance (TSR = 77% and 75%), lower conditioned ITS (355.7 and 337.7 kPa), and higher rut depths (~13.0 and 14.2 mm), making them less suitable for high-stress pavement layers. These findings support Malakand and Kohat aggregates as viable regional alternatives to Margalla.

## 1. Introduction

Natural aggregates are the most commonly used material in highway construction, making up over 90% of the total weight in asphalt pavements. Because of this, the overall quality of an asphalt pavement depends greatly on the quality of the aggregates [1]. This quality is influenced by factors like shape, surface texture, and sharpness of edges [2]. Shape characteristics affect how flat or elongated the coarse particles are. The angularity and particle index help determine how well the aggregates fit together [1]. These shape features also affect how the asphalt mixture handles repeated traffic loading. This effect becomes more noticeable with thicker pavement layers.

To improve the strength, durability, safety, and performance of asphalt mixtures, the Strategic Highway Research Program (SHRP) introduced the Superpave system in 1993. Superpave brought major improvements in how asphalt binders are classified and how asphalt mixtures are designed. It also defined specific standards for aggregate quality, known as consensus and source properties [3]. SHRP researchers identified four key consensus properties that directly impact pavement performance: the angularity of both coarse aggregates, the amount of flat and elongated particles in coarse aggregates, and the sand equivalent value of fine aggregates.

Pan et al. [4] found a strong link between the aggregate properties outlined by SHRP and the mechanical performance of asphalt mixtures. Similarly, Aragão et al. [3] showed that coarse aggregate characteristics had a better relationship with damage resistance. The researchers also noted that the surface texture of the particles had a strong effect on asphalt performance and suggested that it should be included in the mix design standards. Hu et al. [5] observed that larger particle sizes led to more deformation in the asphalt mastic. Aggregate shape has also been recognized as a key factor affecting asphalt performance. Many studies have looked into how different shape parameters influence results. They found that angular and cubical aggregates perform best [6,7]. Mixtures with flaky aggregates require more asphalt binder and tend to have lower resistance to permanent deformation and lower resilient modulus. On the other hand, cubical aggregates improve resistance to creep and increase resilient modulus [8]. Rough surface texture can also improve the bond between aggregate and binder, reducing the chance of fatigue cracking [9]. Studies have further confirmed that angularity and texture play a major role in rutting and skid resistance. Flat and elongated aggregates are generally not preferred because they deter compaction and cause more breakage of coarse particles during construction. Recent studies have emphasized the importance of integrating macro-scale engineering properties with microstructural characterization to better understand material performance. Microstructural features, including morphology, mineral composition, and surface texture, have been shown to significantly influence the mechanical behavior and durability of construction materials. Advanced characterization techniques provide valuable insights into the relationship between material structure and performance, enabling more reliable and sustainable material selection for infrastructure applications. Therefore, combining conventional engineering tests with micro-level analyses can facilitate a comprehensive performance-based evaluation of pavement materials [10].

The Federal Highway Administration (FHWA, 2000) [11] studied the effects of flat and elongated aggregates on asphalt specimens made with a gyratory compactor. They used two types of aggregates: gravel and dolomite. For gravel, the flat and elongated particles created more air voids because they could not easily move or settle into place. In the case of dolomite, which is softer than gravel, the shape caused more breakage, shifting the particle sizes and changing the overall gradation of the mix. Another study using the same compaction method found that mixes with more flat and elongated particles needed more effort to reach the required air voids. Asphalt mixes with flat and elongated particles also tend to have lower density and are harder to compact properly, leading to poor workability compared to mixes with more cubical aggregates [8]. The shape of the aggregates not only affects compaction and density but also has a major influence on mechanical properties such as rutting resistance, skid resistance, moisture resistance, and both resilient and stiffness moduli. Other researchers have emphasized that during both compaction and actual use, the physical features of the aggregates like size, shape, and angularity play a critical role in performance [12]. Aragão et al. [3] also showed that the stiffness modulus, especially at lower loading speeds or frequencies, is influenced by how the aggregate particles are shaped.

Recent advancements in aggregate morphology characterization have increasingly relied on advanced imaging technologies and automated image-analysis methods. Peng and Yang [12] utilized X-ray CT images combined with convolutional neural network (CNN)-based segmentation to accurately identify aggregate boundaries within asphalt mixtures. Zhang et al. [13] employed X-ray computed tomography (XCT) and computational geometry techniques to quantify multi-scale aggregate morphological descriptors and evaluate their interrelationships. Similarly, studies on porous asphalt mixtures utilized XCT-based three-dimensional reconstruction to investigate the influence of aggregate morphology on pavement structural characteristics. In 2025, Ren et al. [14] developed an image-based evaluation framework using digital image processing to assess aggregate shape quality in stacked aggregate systems, while other researchers employed machine-vision technologies and aggregate image measurement systems (AIMS) for quantitative characterization of angularity, texture, and shape indices. More recently, Moghadam et al. [15] proposed a photogrammetry-based three-dimensional reconstruction approach and a field computer-vision framework for comprehensive aggregate morphology characterization, providing cost-effective alternatives to laser scanning and XCT methods. These developments demonstrate the growing application of advanced imaging techniques for objective and quantitative evaluation of aggregate angularity and morphology, supporting their integration into pavement material performance assessment.

Careful selection of aggregates according to defined standards can significantly improve pavement performance and reduce early damage. Aggregates used in roads and highways must resist wear and tear, particularly from abrasion. The performance of such aggregates largely depends on their source and the crushing methods used during processing. Pakistan is rich in limestone resources, especially from intact rock, and these are commonly crushed for use in construction. One of the main sources of aggregate in Punjab and Khyber Pakhtunkhwa is the Margalla Hills limestone. This material has been widely used by the National Highway Authority (NHA), including in major motorway projects across Pakistan. However, the rapid growth in population and infrastructure development has led to increasing demand, putting pressure on existing aggregate reserves. Pakistan’s road network covers about 264,175 km, connecting both national and regional routes [16]. These roads are crucial to the country’s economy, as they carry nearly 96% of all inland freight [17]. Recognizing this, the NHA has launched several strategic projects, including building new roads and upgrading older ones, leading to even higher demand for aggregates [18]. Consequently, Margalla aggregates are depleting with time. This makes it critical to evaluate other potential sources to reduce overreliance on Margalla quarries. Several studies have been conducted to examine alternative aggregate sources. Ahmad et al. [19], for example, reported that aggregates from Thandyani, Muzaffarabad, and Swat did not meet standards for tests like Los Angeles Abrasion, flakiness, elongation, water absorption, and specific gravity. Another study recommended aggregates from Dina as a suitable alternative to Margalla [20]. Still, there is a pressing need to explore and test more quarries across Pakistan to meet the growing construction demands nationwide.

### 1.1. Research Significance

**Figure 1:**
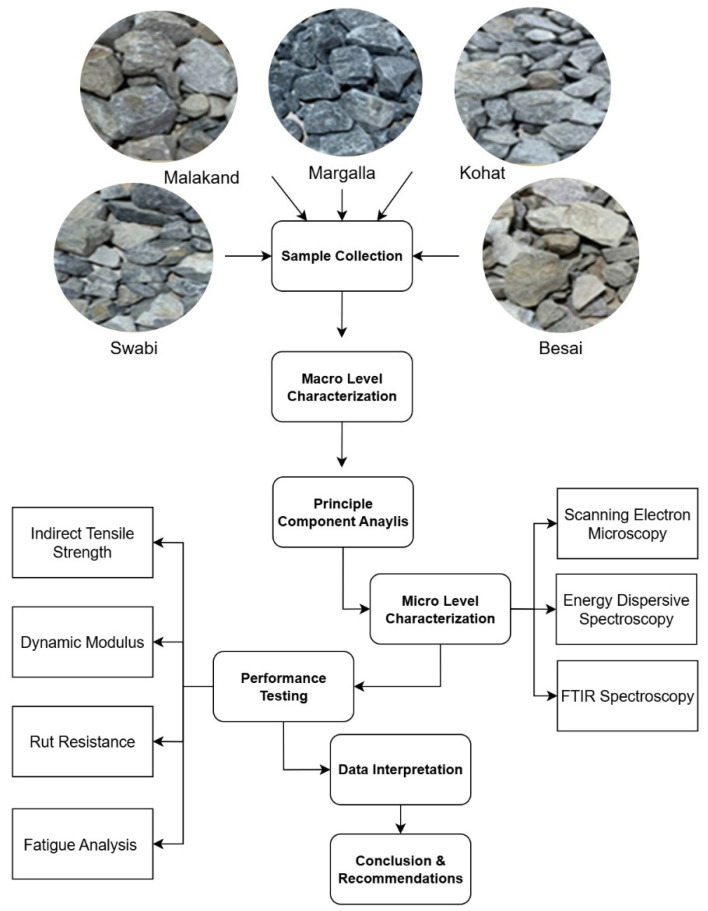
Methodological flowchart.

Flexible pavements are the most commonly adopted pavement type across the globe and typically consist of up to 95% aggregates by volume. These aggregates are the key components in the structural integrity, durability, and long-term performance of these pavements. Consequently, it is necessary to select aggregates with sufficient strength and durability. In Pakistan, the aggregates sourced from the Margalla Hills quarry are regarded as the most suitable aggregates for highway construction. However, due to the growing demand for infrastructure development, these aggregates are depleting, necessitating the evaluation of new sources to alleviate strain on Margalla aggregates. Moreover, relying on a single quarry is not feasible for meeting the aggregate demand across the entire province, as long transportation distances increase both project costs and construction time. To address this issue, the current study is focusing on the evaluation of aggregates sourced from five different quarries based on macro- and micro-levels. Both macro- and micro-level analyses are conducted to assess their suitability for use in flexible pavement applications. A comprehensive set of laboratory tests, in accordance with standard procedures, is performed on asphaltic samples to evaluate the mechanical and morphological characteristics of the aggregates. The results are compared against those of Margalla aggregates, providing insights into alternative aggregate sources that can reduce reliance on a single quarry and support sustainable pavement construction. The methodology adopted for the current study has been presented in the Figure 1.

### 1.2. Research Hypothesis

Due to the diminishing availability of aggregate in the Margalla region of Punjab, and transportation delays for materials shipped across provincial borders, it is proposed that local aggregate from Khyber Pakhtunkhwa (KP) can be a suitable replacement for asphalt mixture production, so long as the performance and durability requirements are equivalent or better. To accomplish this, five quarries, including sources from KP (i.e., Malakand, Kohat, Swabi, and Besai) were studied through an exhaustive characterization of their physical and mechanical properties by utilizing common aggregate tests (i.e., abrasion, flakiness, impact value) and full mineralogical and chemical analyses using SEM, FTIR, and EDS. This multiple-pronged approach can identify the best available and regionally compliant aggregate while achieving sustainable, affordable, and efficient infrastructure development and implementation in KP.

### 1.3. Sustainability Considerations

In addition to technical performance, this study considers the sustainability implications of utilizing regional aggregate sources within Khyber Pakhtunkhwa (KP) Province. The results indicate that Malakand and Kohat aggregates exhibited satisfactory engineering performance, with TSR values of 79% and 76%, conditioned ITS values of 408.7 and 377.7 kPa, and relatively low rut depths compared to Swabi and Besai aggregates. These findings demonstrate that selected regional aggregates can serve as viable alternatives to Margalla aggregates for flexible pavement applications. From a sustainability perspective, the use of locally available aggregate resources can reduce reliance on the increasingly depleted Margalla quarry, promote the efficient utilization of regional natural resources, and potentially decrease transportation requirements associated with aggregate supply. Furthermore, the SEM, FTIR, and EDS analyses confirmed the favorable mineralogical and chemical characteristics of the regional aggregates, supporting their suitability for durable pavement construction. Improved pavement durability can contribute to sustainability by reducing the frequency of maintenance and rehabilitation activities, thereby conserving materials and energy over the pavement life cycle. Although a detailed environmental or life-cycle assessment was beyond the scope of this study, the findings highlight the potential of regional aggregate utilization to support more sustainable and resource-efficient pavement infrastructure development.

## 2. Materials and Methods

The pavement structures are designed and constructed to sustain the loading and provide adequate serviceability. The researchers focus on the different properties of asphalt and its modification to enhance the service life. The performance of the asphalt mixtures focuses on the morphological properties of the aggregates.

### 2.1. Aggregate

**Figure 2:**
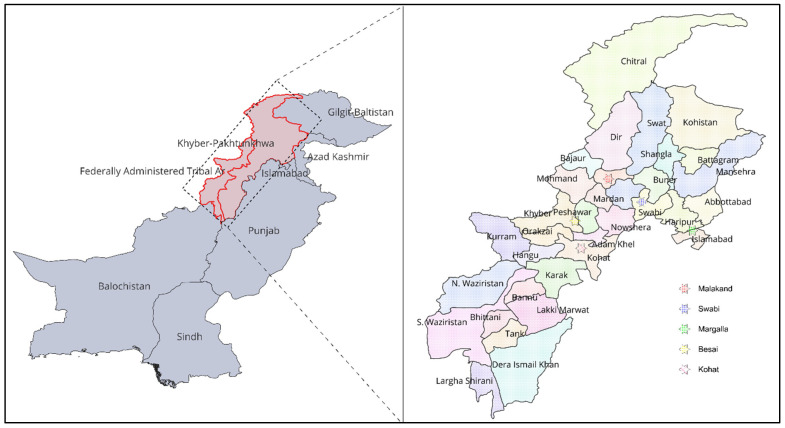
Aggregates source locations [21].

The study is focused on evaluating the suitability of aggregates acquired from five different quarries. A significant amount of crushed aggregates were collected from the quarries including the Swabi, Margalla hill, Malakand, Kohat and Besai quarry. While collecting the samples, a perfect blend of course, fine, and fillers was acquired, and a quartering approach was adopted to ensure the homogeneity of aggregates. A brief overview of the location of the selected quarries has been presented in Figure 2.

### 2.2. Asphalt Binder

Considering the environmental conditions of Khyber Pakhtunkhwa, Pakistan, the asphalt binder of 60/70 penetration grade was sourced from the Attock Refinery in Attock, Pakistan. Physical characteristics of the binder were double-checked in the lab before application, following the ASTM standards. The properties of the binder observed in the lab can be observed in the Table 1.

### 2.3. Gradation and Mix Design

**Figure 3:**
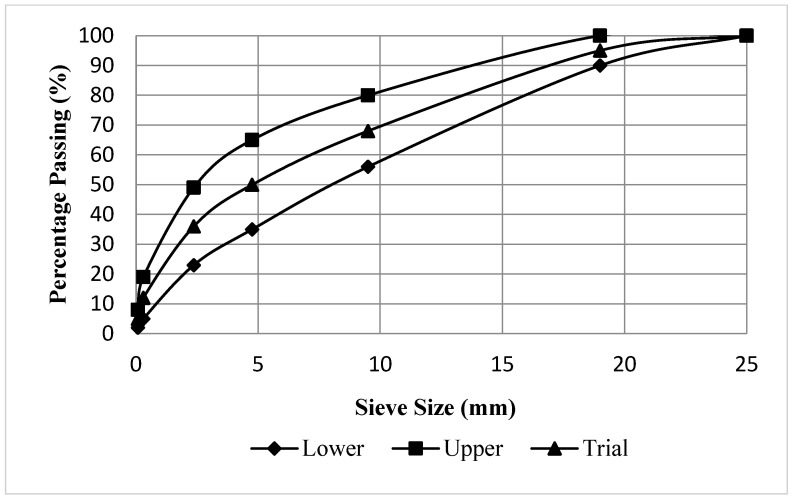
Aggregate gradation.

The performance of asphaltic mix is highly influenced by the gradation and bitumen to aggregate ratio. Hence it is very important to calculate the optimum quantity of every aggregate size and optimum bitumen content (OBC) in the overall mix [27]. In this regard, the aggregate gradation, as presented in Figure 3, was selected based on the limits defined by the National Highways Authority (NHA) of Pakistan. OBC was calculated by testing a series of Marshall samples using aggregates from every quarry. A set of three replica Marshall samples of size 101.6 mm in diameter and 63.5 mm in height were tested for six bitumen content varying between 3.5 and 6% by weight. However, it is worth mentioning that the OBC for every quarry was different due to the different set of aggregates in the mix.

The volumetric specifications, including VMA (Voids in Mineral Aggregate), VFA (Voids Filled with Asphalt), and AV (air voids), of the various mixtures of the asphalt mixtures for the five types of aggregate (Margalla, Malakand, Kohat, Swabi, and Besai) using NHA Class A gradation, which were prepared at their respective binder contents, are outlined in Table 2, and the specification limits are presented in Table 3. Margalla aggregates produced the best volumetric specifications with moderate VMA (15.5%), high VFA (76.5%), and low air voids (3.65%). The mixture produced using Margalla aggregates was a dense and durable mix. Aggregates from Malakand, Kohat, Swabi and Besai produced progressively lower specifications with decreasing aggregate quality, but all the aggregates exhibited a smooth trend of increasing VMA and AV with decreasing VFA. Malakand and Kohat aggregates were rated very good and good, respectively, with an acceptable aggregate AV, though Kohat had a slightly elevated air voids specification (5.00%). Swabi and Besai aggregates were rated good and poor, respectively, and had excess maximum binder content but both also had high AV (5.40% and 6.20%, respectively), indicating low relative compaction and durability concerns, and reduced VFA. Margalla and Malakand aggregates had higher dust-to-binder ratios, and therefore, they kept within the recommended dust-to-binder range of 0.6 to 1.6 (SuperPave guidelines) and 0.6 to 1.2 (NHA guidelines) for the maximum dosage of binder that accounts for all aggregates. The dust-to-binder ratio was determined as the ratio of the percentage of aggregate passing the 0.075 mm (No. 200) sieve to the optimum total asphalt binder content (OBC), according to Equation (1).

Dust-to-Binder Ratio = P_0.075_/P_b_ (1)

where P_0.075_ is the percentage of aggregate passing the 0.075 mm (No. 200) sieve (mineral filler content) and P_b_ is the optimum asphalt binder content by total mixture weight.

However, Besai and Swabi had lower dust-to-binder ratios and therefore did not meet the specification limits. The trends observed across these aggregates illustrated the importance of aggregate quality in achieving desirable performances in mixtures and meeting specifications by the NHA.

The Marshall design limits used are presented in Table 3.

### 2.4. Macro-Level Characterization

**Figure 4:**
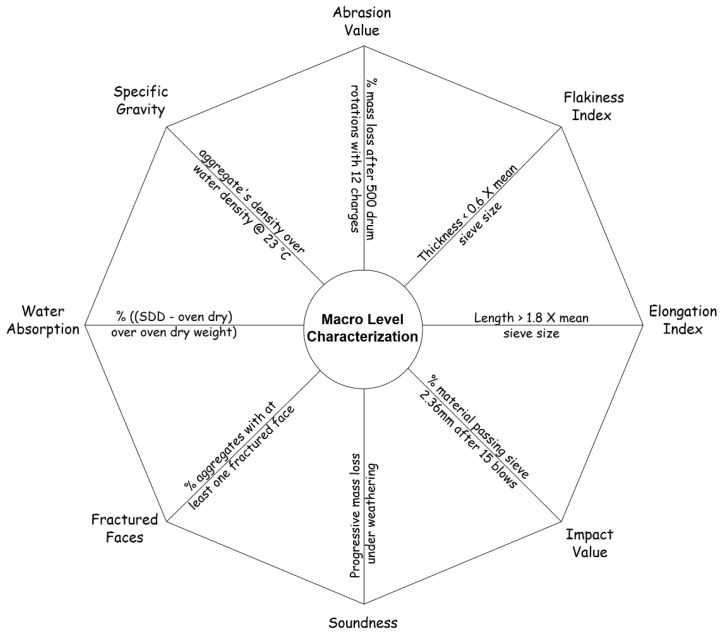
Macro-level performance testing.

Physical properties have a huge impact on the structural performance of highway facilities. Such properties directly impact the air voids, bitumen absorption, resistance to moisture damage, bonding, skid resistance, resistance to wear and tear, dynamic stability, etc. [28,29]. Properties such as resistance to abrasion increase the structural strength, load-bearing capacity, rut resistance, etc.; water absorption governs moisture-induced damage and affects the optimum binder content. Aggregate’s angularity and roundness have an impact on its bonding, stripping, and mix stability. The current study considered various tests like the abrasion value test, soundness, aggregate impact value test, flakiness, and elongation index, etc., for the aggregates sourced from all the quarries. The list of adopted tests for macro-level characterization has been presented in the Figure 4. Standard procedures were followed while studying the physical properties of aggregates.

### 2.5. Micro-Level Characterization

The conventional physical property tests provide essential macro-scale data on aggregate strength, porosity, absorption, etc. However, they don’t explain the hidden patterns and structure of the aggregates for mix optimization. This is why the current study included studying the properties of aggregates on the microscale alongside the macro-scale. In this regard, the following testing procedures were adopted for the aggregates from all the selected quarries.

#### 2.5.1. Scanning Electron Microscopy

**Figure 5:**
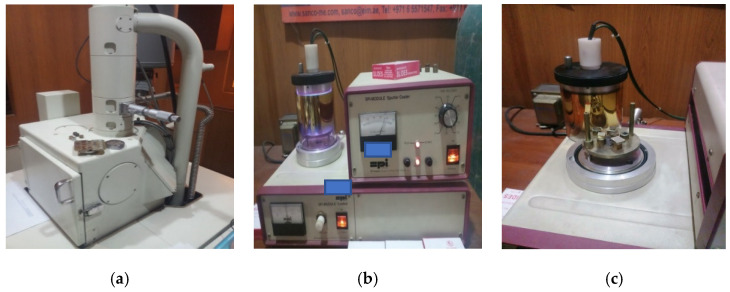
Scanning Electron Microscopy: (**a**) SEM set-up, (**b**) gold coater, and (**c**) sample coating [31].

Scanning Electron Microscopy (SEM) is often adopted to study the microstructural changes in materials [30]. In this study, the SEM JEOL JSM IT 100, featuring Energy Dispersive X-ray Spectroscopy (EDS), Backscattered Electron Detector (BSED), and Secondary Electron Detector (SED), was used with magnifications from 100 to 30,000, as illustrated in Figure 5. Aggregate specimens were first sliced with a diamond saw and then cut into 10 × 10 × 6 mm and 8 × 8 × 6 mm pieces. To make the surfaces of aggregates conductive, the samples were coated with a 4 nm thick gold film using a Gold Sputter Coater as shown in Figure 5. The thin gold coating preserved micropatterns on the samples, enabling detailed SEM analysis to investigate and record the material’s structure.

#### 2.5.2. Energy Dispersive Spectroscopy

Energy Dispersive Spectroscopy (EDS) helps in studying the elemental composition of aggregates. Understanding the elemental composition of aggregates is essential, as the presence of various harmful minerals may affect the mechanical strength of the overall mix. The minerals, such as reactive silica, sulfate, or chloride-rich minerals, may lead to alkali silica reaction, sulfate attack, or corrosion of steel if embedded [32,33]. Additionally, EDS helps in selecting the aggregates with minimal deleterious substances [34]. In the current study, EDS was done using SEM JEOL JSM IT 100 for aggregate samples collected from different quarries. EDS, which is frequently combined with Scanning Electron Microscopy (SEM), picks up X-rays that are released from a sample after it is exposed to an electron beam. As each element emits X-rays at different energies, the elements present can be identified both qualitatively and quantitatively. In material science, this method is particularly useful for examining metals, minerals, and composite materials since it can provide information on surface composition, contamination, or structural differences at the microscale.

#### 2.5.3. Fourier Transform Infrared Spectroscopy

Fourier Transform Infrared Spectroscopy (FTIR) plays a key role in evaluating the potential of testing material in various applications. Additionally, it helps in identifying the mineralogical nature of the aggregates by identifying the presence of carbonates, silicates, quartz, feldspar, etc. The presence of such elements can affect the aggregate’s performance in asphalt or concrete applications [35,36]. In the current study, the molecular structure and chemical composition of aggregate samples were determined using FTIR based on their infrared absorption properties. Instead of looking for individual elements, FTIR looks for certain functional groups and molecular bonds, in contrast to elemental analysis techniques. The light that a sample absorbs when exposed to infrared radiation is at frequencies that match the vibrations of its chemical bonds [37]. Organic molecules, polymers, and certain inorganic materials can be identified using the resultant spectrum, which acts as a molecular fingerprint. Quality control, material characterization, and contaminant analysis all extensively utilize FTIR.

### 2.6. Mechanical Testing

#### 2.6.1. Rutting Test Using Wheel Tracking Machine

**Figure 6:**
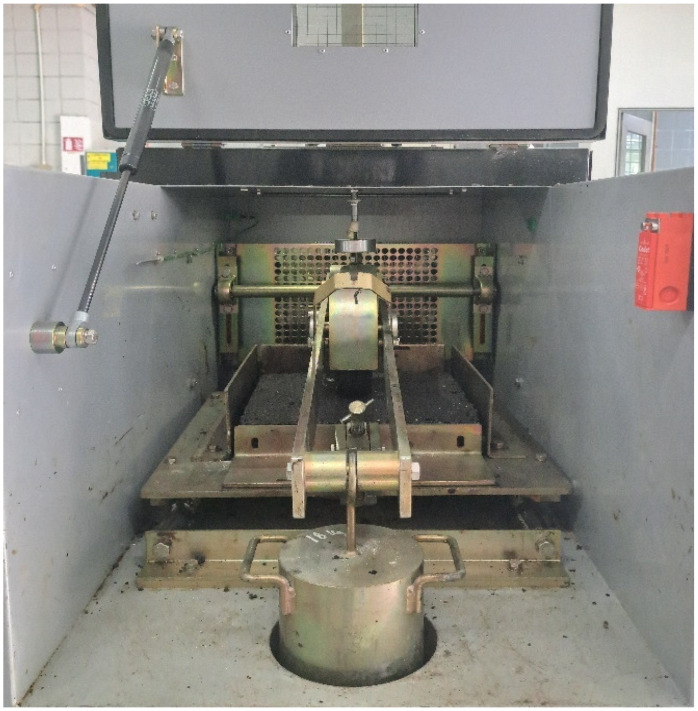
Wheel tracking machine for rutting depth evaluation.

One of the critical asphalt mixes performance requirements is resistance against permanent deformation [38]. In this research work, the Wessex Wheel Tracking (Model S867, Wessex Test Equipment, Banwell, Weston-super-Mare, North Somerset, UK) device, as shown in Figure 6, was utilized to evaluate the resistance to rutting following BS 598–110 standard [39]. The asphalt mixtures’ ability to withstand permanent deformation under repeated loading was assessed using the rutting test. Using a wheel tracking machine, asphalt samples are put through 10,000 passes of a loaded wheel at a regulated temperature of 55 °C. This high temperature mimics extreme summer weather, which makes asphalt more prone to deformation. To evaluate the material’s resistance to rutting, the depth of the rut that forms on the sample surface is evaluated. Better performance and increased resistance to long-term deformation under traffic stress are indicated by a reduced rut depth.

#### 2.6.2. Moisture Susceptibility Test

One of the major causes of distress in the asphalt pavement layers is damage caused by moisture in asphalt mixtures, and the assessment of moisture damage is very critical since it directly impacts pavement performance and service life [40]. With respect to this, the Indirect Tensile Strength (ITS) of bituminous mixes can be calculated in order to evaluate the resistance of bituminous mixes to moisture damage by calculating Tensile Strength Ratio (TSR) of the bituminous mixes using the ITS before and after moisture damage. Various studies have indicated that bituminous mixes with TSR greater than 80% can provide good resistance to moisture damage [41,42]. The current study adopted the procedure followed by Wang et al. [43] and, while calculating the ITS for aggregate acquired from various quarries, Islam et al. [44]. The load was applied on diametrical axis at a rate of 50.8 mm/min until specimen failure in accordance with ASTM D6931-17 [42]. Two sets of three Marshall samples were prepared for the aggregates of every quarry. One set of specimens was conditioned in a water bath at 90 °C for 24 h, and then the samples were transferred to another water bath at 25 °C for 30 min. The ITS for this set was noted as ITS_wet_. The other set of samples was conditioned at 25 °C for 30 min, later referred to as ITS_dry_. The ITS values were calculated using expression given in Equation (2), while the TSR was calculated by dividing the ITS wet with ITS dry as shown in Equation (3).

(2) $$ITS=\frac{2000\times P}{\pi \times t\times d}$$

(3) $$TSR=\left[\frac{{ITS}_{wet}}{{ITS}_{dry}}\right]\times 100$$

where P is peak load, t is thickness, and d is diameter of the sample.

#### 2.6.3. Dynamic Modulus Test

**Figure 7:**
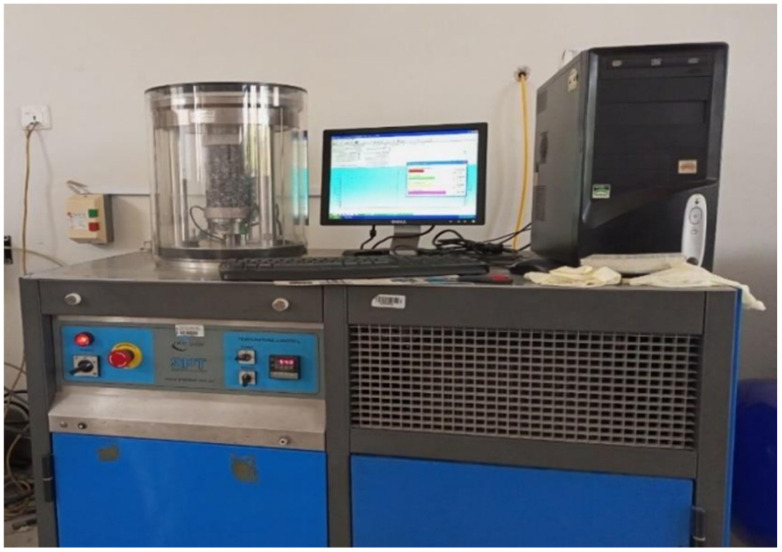
Simple performance tester for dynamic modulus evaluation.

The uniaxial compression dynamic modulus of asphalt mixtures plays a critical role in asphalt pavement design worldwide, as it enhances the precision of structural analysis and modeling [45]. The dynamic modulus (E*), defined as the absolute value of a complex quantity, measures the stiffness of hot mix asphalt (HMA) under sinusoidal loading conditions. It characterizes the stress–strain relationship of linear viscoelastic materials subjected to cyclic loading. The real part of the dynamic modulus represents the elastic stiffness, while the imaginary part corresponds to the material’s internal damping or viscosity [46]. Accordingly, Equation (4) can be used to express both the real and imaginary components of the dynamic modulus (E*). In the current study, the dynamic modulus of the bitumen mixture prepared with aggregates sourced from various quarries was assessed using an asphalt mixture performance tester as shown in Figure 7. The viscoelastic behavior of bituminous mixes was measured at different frequencies like 0.1, 0.5, 1, 10, and 25 Hz at 54 °C. The testing was conducted at a high temperature as asphaltic pavements can experience high temperatures such as 50 °C and above [47,48].

(4) $$E^{*}=E^{\prime }+iE^{\prime \prime }$$

where i = Imaginary unit, defined as √−1, E″ = loss or viscous modulus, and E′ = storage or elastic Modulus.

## 3. Results and Discussion

### 3.1. Macro-Level Characterization

The mechanical and physical properties of aggregates from five different quarries, Margalla from the Punjab province and Malakand, Kohat, Swabi, and Besai from the Khyber Pakhtunkhwa province, were evaluated to determine their suitability for use in flexible pavements. The Los Angeles abrasion value, which measures resistance to wear, was found to be lowest for Margalla (21%) and highest for Besai (33.7%) as shown in Table 4. According to ASTM C131 [49], the permissible limits are ≤30% for base courses and ≤35% for sub-base courses, indicating that all aggregates except Besai are suitable for base layers, while Besai is better suited for sub-base applications. On the other hand, the flakiness and elongation indices ranged from 4.2% to 14.4% and 4.6% to 14.3%, respectively. Margalla again performed the best, indicating more cubical and better-shaped particles. All quarries met the BS 812-105 standards [50], which specify maximum limits of 25% and 30% for flakiness and elongation indices, respectively. These results confirm that aggregates from all sources have acceptable shape characteristics, with Margalla showing superior quality.

Impact resistance, evaluated through the loss in impact value test, varied from 5.7% (Margalla) to 9.4% (Besai). These values are well below the BS 812-3 [51] limits of ≤30% for wearing courses and ≤40% for base layers, indicating that all aggregate sources have adequate toughness and can withstand heavy traffic loads. The degree of unsoundness, which assesses resistance to weathering, was highest in Besai (11.2%) and lowest in Margalla (5.2%), all within the ASTM C88 [52] limit of 18%. This demonstrates that all quarries provide durable aggregates for long-term pavement performance. Fractured face values were above the 90% threshold (ASTM D5821 [53]) for all sources, with Margalla achieving the highest percentage (99.3%), highlighting excellent interlocking capability which is essential for high-performance pavements. Water absorption rates ranged from 0.38% (Margalla) to 0.94% (Besai), all within the ASTM C127 limit of ≤2.0%. Lower absorption suggests reduced moisture susceptibility and better durability and again places the Margalla aggregates at the top. Specific gravity values ranged between 2.57 and 2.89, fitting in the acceptable range of 2.5–3.0 as per ASTM C127 [54]. Margalla aggregates exhibited the highest density, indicating better compaction and structural strength.

Conclusively, Margalla aggregates outperformed other quarries in all parameters, making them the most suitable for high-quality pavement construction. Nonetheless, aggregates from Malakand, Kohat, and Swabi also met all standard requirements and may serve as viable alternatives, especially when considering cost or transport constraints. Besai aggregates, while marginally substandard, still met the minimum standards and can be utilized in less critical pavement layers.

#### 3.1.1. Principle Component Analysis (PCA) for Quarry Aggregate Properties

The Principal Component Analysis (PCA) was conducted to evaluate the variability in quarry aggregate properties and to identify key factors influencing quality. It should be noted that the PCA results are based on a limited dataset comprising five quarry sources. Therefore, PCA is utilized as an exploratory and supporting ranking technique to evaluate the relative performance of the investigated aggregates rather than as a statistically broad classification model. The findings were interpreted within the scope of the selected quarry sources and used in conjunction with conventional engineering property assessments and normalized scoring analyses. The PCA ranking results are summarized in the revised Table 5 and Table 6.

The PCA ranking is consistent with the normalized scoring results, with Margalla exhibiting the highest overall performance and Besai the lowest. Since PC1 explains approximately 95.96% of the total variance, it adequately captures the dominant performance differences among the investigated aggregate sources.

#### 3.1.2. Normalized Scoring and Ranking

Since the variables are expressed in different units, the data was standardized and normalized to allow performance comparison. The direction of improvement for each property was defined as follows: lower-is-better (abrasion, flakiness, elongation, loss in impact, unsoundness, water absorption) and higher-is-better (fractured faces, specific gravity). Equations (5) and (6) were adopted to calculate the normalized score for lower-is-better and higher-is-better properties, respectively. For the normalized results, 1 stands for the best while 0 stands for the worst results as presented in Table 5.

(5) $$Normalized\;Score\;\left(Lower\;better\right)(Si)=\frac{XMax.-Xi}{XMax.-XMin.}$$

(6) $$Normalized\;Score\;\left(Higher\;better\right)(Si)=\frac{Xi-XMin.}{XMax.-XMin.}$$

where Si is the normalized score, Xi is the observed value, and XMax. and XMin. are the maximum and minimum values of the corresponding property.

The normalized scores for the ranking of quarries are reported in Table 7.

The total score results given in Table 5 differentiate the performance levels of the five queries. Margalla, with a highest score of 8.00, demonstrates excellence across all tested parameters, making it the most reliable source for high-quality aggregates. Malakand, scoring 5.79, ranks as a strong second choice, performing well in most categories but showing noticeable limitations in flakiness and elongation indices. While still suitable for many structural applications, these shape-related deficiencies could influence compaction efficiency and interlocking behavior in pavements. The performance of the aggregates from various quarries has been interpreted based on the above analysis and presented in Table 8.

Based on the macro-level characterization, Margalla stands out as the best-performing option and should be the preferred choice when quality is the first priority. In situations where Margalla is not available, Malakand serves as a viable alternative due to its comparatively acceptable performance. However, Besai should be avoided, especially in critical applications, due to its lower quality characteristics.

### 3.2. Micro-Level Characterization

#### 3.2.1. Scanning Electron Microscopy

**Figure 8:**
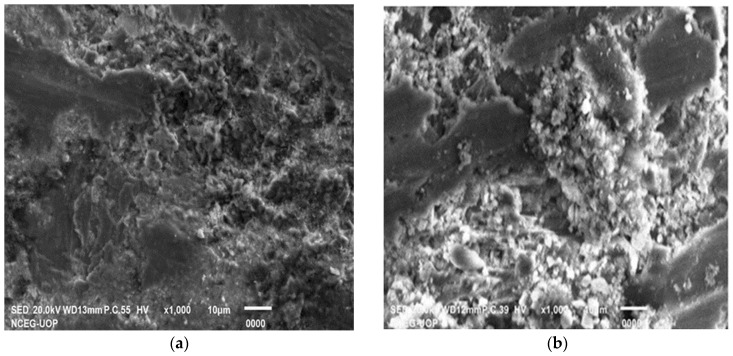

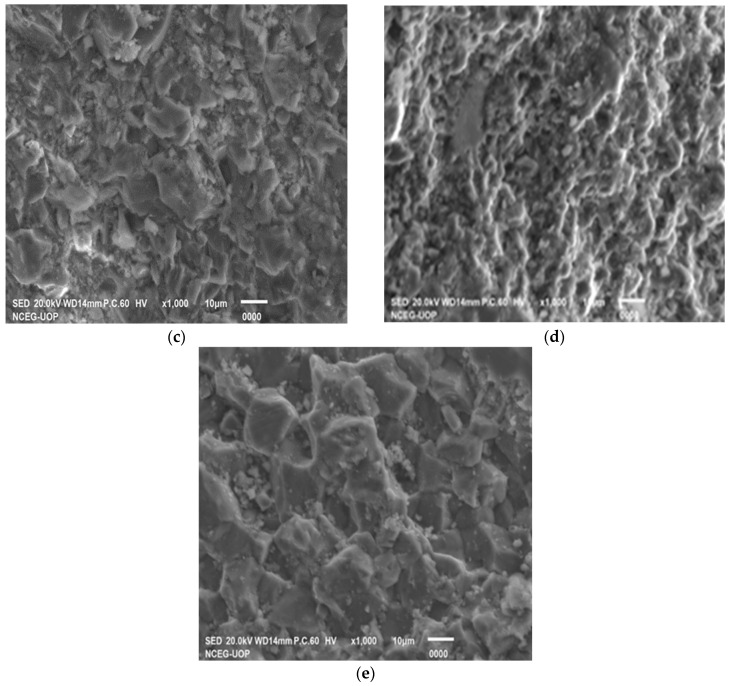
SEM images: (**a**) Margalla aggregate, (**b**) Malakand aggregate, (**c**) Swabi aggregate, (**d**) Kohat aggregate, and (**e**) Besai aggregate.

Scanning Electron Microscopy (SEM) was employed to examine the surface morphology and microstructural features of aggregates from five quarries: Margalla, Malakand, Swabi, Kohat, and Besai (shown in Figure 8) based on 15 samples. These micrographs provide valuable insight into the texture, compactness, and surface roughness, all of which significantly influence the mechanical behavior and durability of aggregates in pavement applications. Margalla aggregate shows a compact and relatively smooth surface with fewer visible pores and microcracks. This dense structure explains its superior performance in mechanical tests, such as lower abrasion and impact values, and minimal water absorption. The compact matrix contributes to higher strength and durability, making Margalla aggregate most suitable for high-stress pavement layers. Malakand aggregate presents a rougher surface with visible granules and a somewhat porous texture. This increased surface irregularity may improve interfacial bonding with binders but can also lead to higher water absorption and slightly reduced durability compared to Margalla. Nonetheless, the microstructure remains sufficiently stable for base and sub-base applications. On the other hand, Swabi aggregate demonstrates a moderately rough surface with irregular particle shapes and interconnected micro-voids. These features suggest lower packing density and higher susceptibility to moisture ingress. Such characteristics correlate with its relatively higher flakiness and water absorption values, indicating limited performance under heavy loading unless properly treated or used in less critical layers. The Kohat aggregate (d) exhibits a notably rugged and fragmented microstructure, with deep fissures and a network of microcracks. This macrotexture indicates reduced structural integrity, which aligns with the comparatively higher abrasion and unsoundness values observed in lab tests. This kind of surface morphology could negatively impact long-term performance, particularly in freeze-thaw or wet conditions. The Besai aggregate (e) on the other hand shows the most porous and irregular microstructure among the samples. The abundance of micro-voids and rough fractured surfaces implies weak structural cohesion, low density, and high water absorption. These features confirm the lower specific gravity and higher impact loss values recorded in the experimental data. While still within acceptable limits for sub-base use, Besai aggregates may require treatment or blending with stronger materials for higher-performance applications.

#### 3.2.2. Energy Dispersive X-Ray Spectroscopy

**Figure 9:**
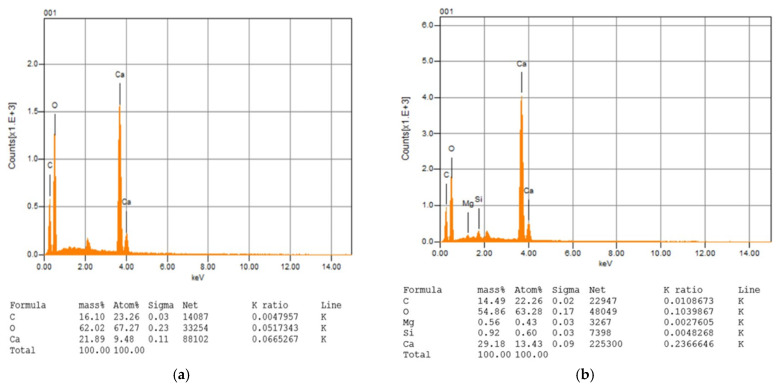

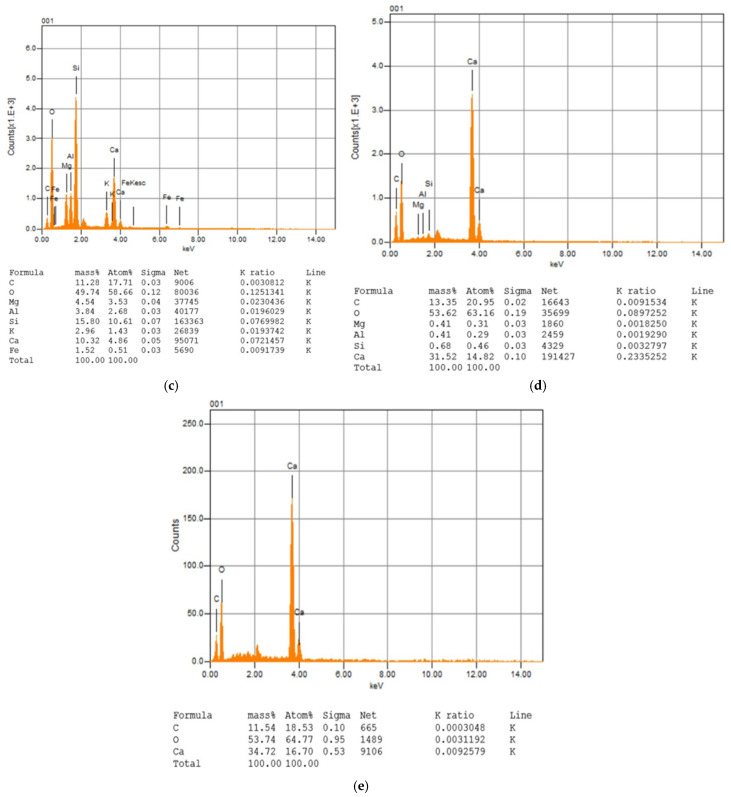
Elemental composition: (**a**) Margalla Aggregate, (**b**) Malakand Aggregate, (**c**) Swabi Aggregate, (**d**) Kohat Aggregate, and (**e**) Besai Aggregate.

Energy Dispersive X-ray Spectroscopy (EDS) reveals the elemental composition, particularly the presence of calcium, silicon, magnesium, and iron, which are critical in determining reactivity and compatibility with cementitious or bituminous matrices [55]. In this study, aggregates collected from five different geological sources in Pakistan including Margalla, Malakand, Swabi, Kohat, and Besai were analyzed using EDS to evaluate their microstructural features and elemental makeup as presented in Figure 9. This characterization is essential to understanding how the origin and composition of aggregates influence their performance in construction applications, particularly in terms of strength, durability, and binder interaction.

By analyzing the Margalla Aggregate (a), the EDS spectrum revealed a composition dominated by calcium at 21.89%, carbon at 16.10%, and oxygen at 62.02%. The high calcium content indicates the presence of calcite (CaCO_3_), typical of limestone geology. This calcium-rich composition is beneficial for strong bitumen-aggregate bonding and explains the excellent mechanical properties. On the other hand, the Malakand aggregate showed a similarly high calcium content (63.21%), with substantial oxygen (21.86%) and carbon (14.49%), further affirming the dominance of calcite or dolomite. The trace presence of Mg and Si suggests minor inclusions of dolomitic limestone or silicate phases. The chemical stability of these constituents supports the durability performance of the aggregate, although marginally lower than that of Margalla due to slightly more textural roughness.

The Swabi aggregate conversely displayed a more heterogeneous composition, including oxygen (49.74%), carbon (11.28%), and notable amounts of magnesium (4.84%), aluminum (4.64%), silicon (3.56%), potassium (2.86%), calcium (11.54%), and iron (1.52%). The presence of silicates, aluminosilicates, and iron-bearing minerals points toward a more igneous or metamorphic origin [56]. These chemically diverse constituents may lead to weaker chemical bonding with cement paste, contributing to lower strength and higher water absorption. Similarly, the Kohat aggregate showed a moderate calcium content (31.52%), with oxygen (53.62%), carbon (13.35%), and traces of Si, Al, and Mg. This composition suggests a mix of carbonates and silicate minerals, typical of calcareous sandstone or partially weathered limestone. While still within acceptable performance thresholds, the lower calcium concentration and mixed composition may affect long-term durability and contribute to marginally poorer performance in mechanical testing. Besai aggregates on the other hand exhibit a calcium concentration of 34.72%, along with oxygen (53.74%) and carbon (11.54%), indicating a dominance of carbonate minerals. However, the lack of significant trace elements (e.g., Mg, Si, Al) and the presence of high porosity, as seen in SEM, suggest a less dense and potentially more reactive limestone. This can explain the higher water absorption and lower specific gravity found in Besai aggregates, which might pose risks for freeze-thaw durability and binder compatibility in harsh environments.

#### 3.2.3. FTIR Spectroscopy

FTIR offers a powerful tool for addressing quality control (QC) and quality assurance (QA) challenges by identifying various chemical components through their infrared (IR) spectral emissions [57]. On the basis of previous published works, carbonate minerals such as calcite and dolomite are typically indicated by a strong asymmetric C-O stretching band in the 1400–1500 cm^−1^ range, an out-of-plane bending mode at 870–880 cm^−1^, and an in-plane bending feature near 700–720 cm^−1^ [58]. These assignments are well established in mineralogical studies and calibration efforts for carbonate abundance. Silicate materials, including quartz and feldspars, display prominent asymmetric Si-O stretching bands between 1000–1100 cm^−1^, with associated lattice or bending vibrations in the 450–500 cm^−1^ region [59]. Moreover, hydroxyl and structural water are identifiable by broad absorbance features between 3400–3600 cm^−1^ and around 1640 cm^−1^, which is consistent with standard clay mineral spectra [59]. Minor peaks in the 600–700 cm^−1^ range are associated with lattice vibrations of silicate clays, iron oxides, or aluminum hydroxide bending modes and have been described in detail for various clay and carbonate systems. Because each bond has a unique vibrational frequency that depends on atomic mass and bond strength, the FTIR spectrum does not exhibit a simple ascending or descending pattern in peak heights or positions; each peak stands as a unique “fingerprint” reflecting the presence and abundance of different mineral groups.

**Figure 10:**
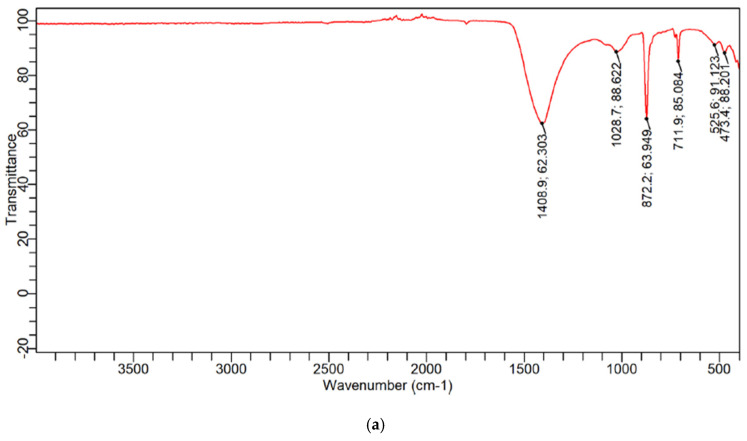

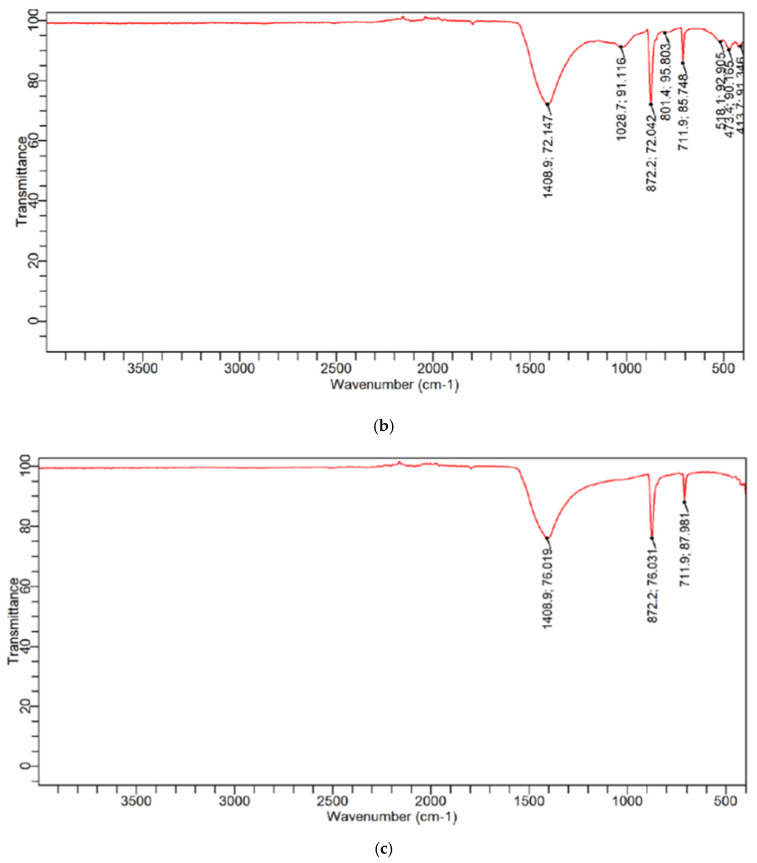
FTIR spectroscopy: (**a**) Margalla Aggregate, (**b**) Malakand Aggregate, and (**c**) Swabi Aggregate.

In the current study, a total of 15 samples were analyzed with FTIR spectroscopy as shown in Figure 10a–c. The FTIR spectrum of Margalla aggregate shows a strong and broad peak at 1408.9 cm^−1^, corresponding to the asymmetric stretching of the CO_3_^2−^ group, indicating the dominance of calcite. Peaks at 872.2 cm^−1^ and 719.9 cm^−1^ represent out-of-plane and in-plane bending modes of carbonate ions, respectively. Additionally, a peak at 1028.7 cm^−1^ is observed, typically related to Si–O stretching, suggesting the presence of silicate impurities or quartz. Multiple low-intensity peaks below 700 cm^−1^ indicate lattice vibrations, reflecting minor clay or aluminosilicate components. Malakand aggregates also showed a strong carbonate peak at 1408.9 cm^−1^, similar to Margalla, suggesting calcite presence. A noticeable difference is the more intense and defined Si–O stretching peak at 1028.5 cm^−1^, along with additional peaks in the 470–520 cm^−1^ range, such as at 476.2 and 458.4 cm^−1^, which imply a higher silicate or clay content compared to Margalla. The 871.2 cm^−1^ and 712.8 cm^−1^ peaks again reflect carbonate bending. The Swabi aggregate shows a sharp carbonate peak at 1408.3 cm^−1^ and bending vibrations at 872.2 cm^−1^ and 711.9 cm^−1^, consistent with calcite. However, this sample lacks a prominent silicate peak around 1020–1030 cm^−1^, unlike the Margalla and Malakand. This implies lower silicate or quartz content. Instead, it shows clearer, sharper carbonate bands, indicating high-purity limestone.

The FTIR results, as shown in Figure 10, provide valuable insight into the mineralogical composition of the aggregates and their influence on asphalt mixture performance. The dominant carbonate peaks observed in all three aggregate sources confirm the prevalence of calcite-rich limestone, which is generally associated with good adhesion to asphalt binders due to its alkaline nature, thereby enhancing moisture resistance and mixture durability. The stronger and more defined silicate-related peaks observed in the Malakand aggregates indicate the presence of quartz and aluminosilicate minerals, which can contribute to higher aggregate hardness and abrasion resistance, resulting in improved resistance to crushing and polishing under traffic loading. Margalla aggregates exhibited a balanced carbonate–silicate composition, which supports both adequate binder affinity and mechanical strength. In contrast, Swabi aggregates showed relatively pure carbonate mineralogy with minimal silicate content, suggesting excellent asphalt–aggregate bonding characteristics but comparatively lower hardness than aggregates containing higher silicate fractions. Overall, the FTIR findings are consistent with the mechanical characterization results, demonstrating that variations in carbonate and silicate mineral content significantly influence aggregate strength, durability, and asphalt mixture performance.

### 3.3. Rutting Potential of Asphalt Mixtures

**Figure 11:**
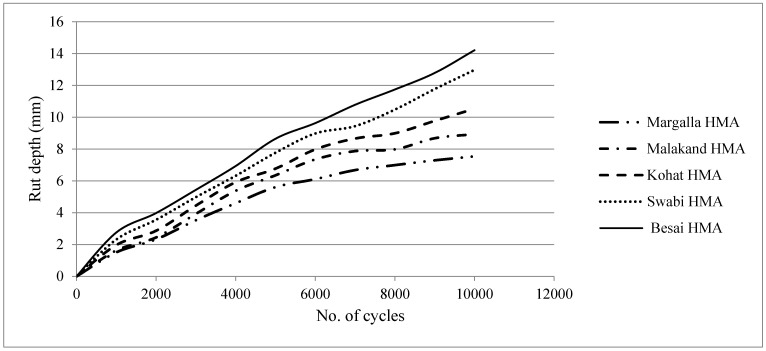
Rut depth against no. of cycles for specified quarries.

The rutting resistance of HMA mixes incorporating aggregates from various quarries and was assessed by subjecting the samples to repeated load cycles up to 10,000 repetitions at 55 °C, as shown in Figure 11. The results clearly indicate a variation in rutting susceptibility among the different aggregate sources. Among all, Margalla HMA exhibited the lowest rut depth (<8 mm) after 10,000 cycles, demonstrating superior rutting resistance. This performance is attributed to the high strength, angularity, and low water absorption of Margalla aggregates, which contribute to better interlock and reduced permanent deformation. Malakand and Kohat HMA also performed well, with rut depths of approximately 8.3 mm and 8.7 mm, respectively. Their relatively better performance may be linked to moderate strength properties and mineralogical composition, as confirmed by SEM-EDS analysis. In contrast, Swabi and Besai HMA mixes exhibited significantly higher rut depths, reaching 13 mm and 14.5 mm, respectively. This poor rutting resistance is attributed to the higher water absorption, lower specific gravity, and greater presence of mineralogical composition, as observed in the SEM microstructure. Aggregates with high water absorption tend to retain moisture, which weakens the bond between the asphalt binder and the aggregate surface, leading to stripping and reduced load-bearing capacity under repeated traffic loads. Similarly, low specific gravity often indicates a porous or lightweight material, which typically lacks the structural strength necessary to resist permanent deformation. Additionally, the relatively lower fractured face percentages and higher flakiness/elongation indices of Swabi and Besai aggregates suggest inferior aggregate interlock, further compromising the structural integrity under repetitive loading. Overall, the results underscore the importance of aggregate quality in determining rutting resistance of HMA. Aggregates with high mechanical strength, angularity, and durability characteristics such as those from Margalla are better suited for applications where resistance to permanent deformation is critical.

### 3.4. Moisture Susceptibility of Asphalt Mixtures

**Figure 12:**
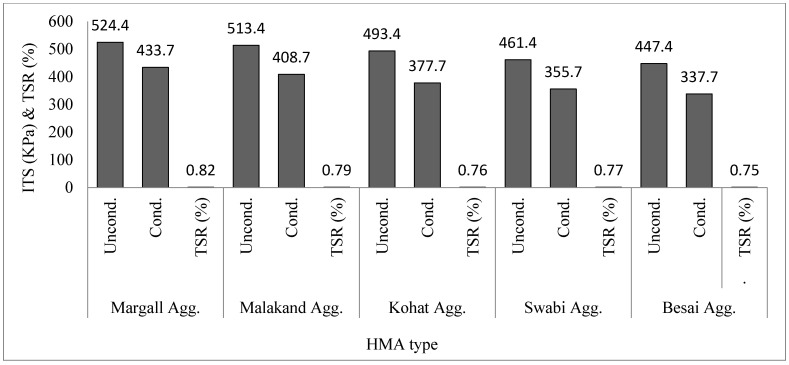
Variation in Indirect Tensile Strength and Tensile Strength Ratio with respect to mix type.

The moisture susceptibility test revealed that the vacuum saturation and freezing-thawing conditioning of samples accelerate the water damage, as shown in Figure 12, by comparing the conditional and unconditional tensile strengths of the different aggregates (Margalla, Malakand, Kohat, Swabi, and Besai) as well as their Tensile Strength Ratio (TSR%). The data shown in Figure 12 illustrates how well each aggregate performs in hot mix asphalt (HMA). A decrease in mechanical performance upon exposure to moisture is indicated by the unconditioned state’s consistently higher values (e.g., 524.4 for Margalla, 513.4 for Malakand) compared to the conditioned state (e.g., 433.7 for Margalla, 408.7 for Malakand). The Margalla aggregate performs the best (satisfying the strength loss retainment requirement of 80% [41]), and the Besai aggregate performs the worst (0.75), according to the TSR values, which measure moisture resistance. The change from a granular to an amorphous state is responsible for this performance change.

The granular and amorphous states of aggregates significantly influence the performance of asphalt mixtures due to their impact on mechanical interlocking, bonding with bitumen, and durability. Granular aggregates, being crystalline and well-defined in structure, typically offer angular shapes and rough textures, which enhance inter-particle friction and promote strong mechanical interlock. This results in improved resistance to deformation (rutting) and better load distribution. In contrast, amorphous aggregates lack a regular crystalline structure and are often smoother and more rounded, leading to reduced friction and weaker aggregate–bitumen bonding. This can diminish the mixture’s stiffness, increase susceptibility to permanent deformation under traffic loads, and potentially accelerate moisture damage due to poor adhesion. Higher tensile strength is the result of aggregates maintaining strong interparticle friction and binder cohesion in the granular state. Conditioning, which mimics prolonged exposure to moisture, alters its structure, weakening the aggregate matrix and softening the binder. This degradation is reflected in the decrease in TSR, which highlights how moisture penetration weakens the amorphous state by decreasing adhesion and raising cracking susceptibility. Because of its innate resistance to moisture-induced degradation, Margalla aggregate exhibits high stability, while aggregate diversity in TSR indicates variations in mineral composition, porosity, and binder affinity. These results highlight how important aggregate selection is to maintaining long-lasting and moisture-resistant asphalt pavements.

### 3.5. Dynamic Modulus of Asphalt Mixtures

**Figure 13:**
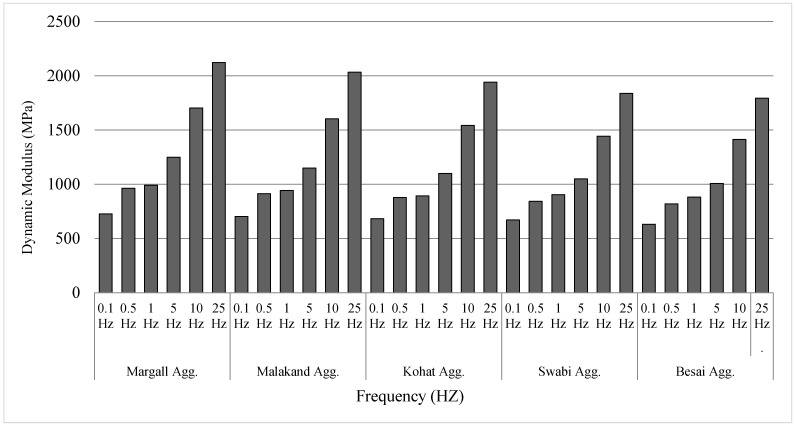
Variation in dynamic modulus with respect to loading frequency and mix type.

Dynamic modulus testing was performed under uniaxial compression using cylindrical asphalt mixture specimens as per AASHTO T 378-17 [60]. The specimens were prepared using a Superpave gyratory compactor and subsequently cored and trimmed to a nominal diameter of 100 mm and a height of 150 mm. Prior to testing, the specimen ends were trimmed to ensure parallel and smooth loading surfaces. The specimens were conditioned at the test temperature of 54 °C before applying cyclic compressive loading at frequencies of 0.1, 0.5, 1, 10, and 25 Hz to characterize the viscoelastic response of the asphalt mixtures. The dynamic modulus, as shown in Figure 13, showed an increasing trend with respect to loading frequencies but a decreasing trend with respect to mix type. The highest dynamic modulus values, particularly at higher frequencies, are found in asphalt mixtures that contain Margalla and Malakand aggregates. Their coarse-grained, crystalline mineralogy, which is frequently linked to igneous or metamorphic rock types like granite or quartzite, might be responsible for this increased rigidity. These aggregates have considerable surface roughness and angular, interlocking particles, which enhance binder adherence and mechanical interlock. They are therefore perfect for high-stress applications due to their enhanced load distribution and resistance to irreversible deformation. By comparing the dynamic modulus of Margalla aggregates with the rest of the quarries, it can be observed that Malakand and Kohat aggregates are closely following the strength of Margalla aggregates at all frequencies. These results were also found to be consistent with the findings of two different studies published by Khan et al. [61].

Swabi and Besai aggregates, on the other hand, have more amorphous or weathered microstructures, which may have been generated from sedimentary rocks like sandstone or limestone. They also have a lower dynamic modulus and more moderate frequency sensitivity. Reduced stiffness but higher flexibility and moderate fatigue tolerance under repeated loading were observed for these aggregates. Because of these features, they can be used on pavements with lower traffic volumes and speeds where fatigue cracking and heat damage are the main issues. With its intermediate stiffness, Kohat aggregate could be a transitional composition with moderate angularity and perhaps a partial crystal structure that offers balanced fatigue and structural performance.

### 3.6. Correlation of Mechanical Properties

**Figure 14:**
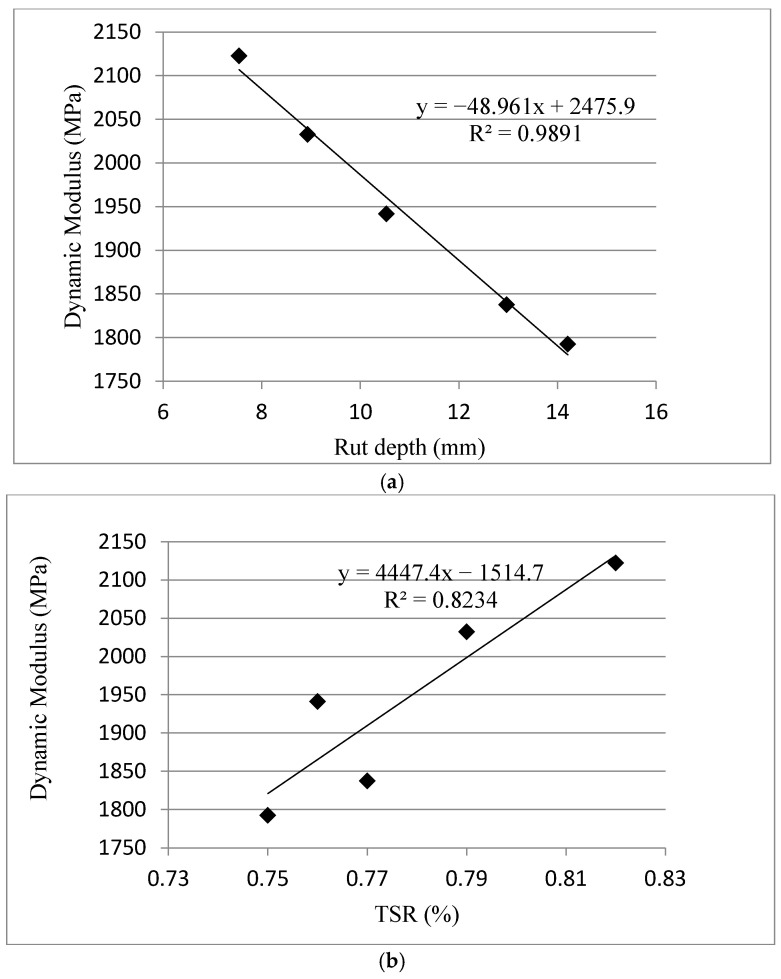

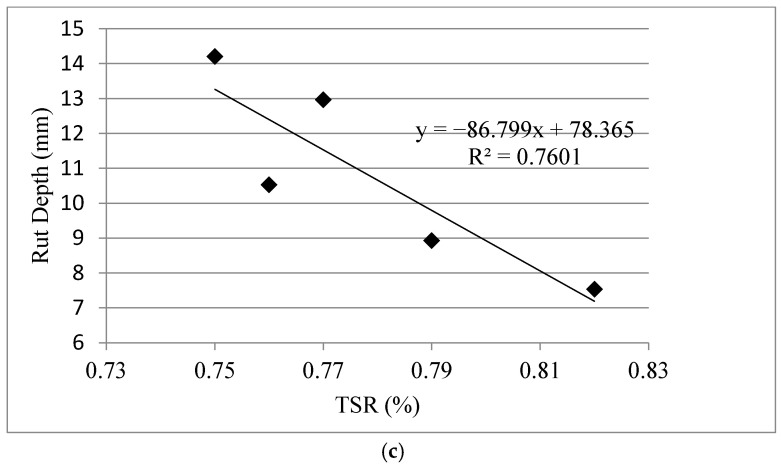
Association among dynamic modulus, rut depth, and percentage TSR.

Figure 14a shows a strong negative correlation between dynamic modulus and rut depth, with an R^2^ value of 0.9891, indicating excellent linear fit. The regression equation, suggests that for around 1 mm increase in rut depth, the dynamic modulus decreases by approximately 48.96 MPa. This result confirms that rutting significantly compromises the structural stiffness of asphalt mixes. Higher rut depths typically result from weaker interlocking and aggregate deformation, which in turn reduces the load-bearing capacity of the pavement. Figure 14b illustrates a positive correlation between dynamic modulus and TSR, with an R^2^ of 0.8234. This implies that moisture-resistant mixes tend to retain higher stiffness, emphasizing the role of good adhesion between aggregates and binder in mitigating moisture-induced damage and preserving mechanical performance. Figure 14c depicts a negative linear relationship between rut depth and TSR, with a moderately strong R^2^ of 0.7601. The regression equation shows that higher moisture resistance (higher TSR) corresponds to reduced rutting depth. Therefore, enhancing moisture resistance through appropriate binder selection or additive use can directly improve rutting performance.

### 3.7. Fatigue Analysis of Asphalt Mixtures

**Figure 15:**
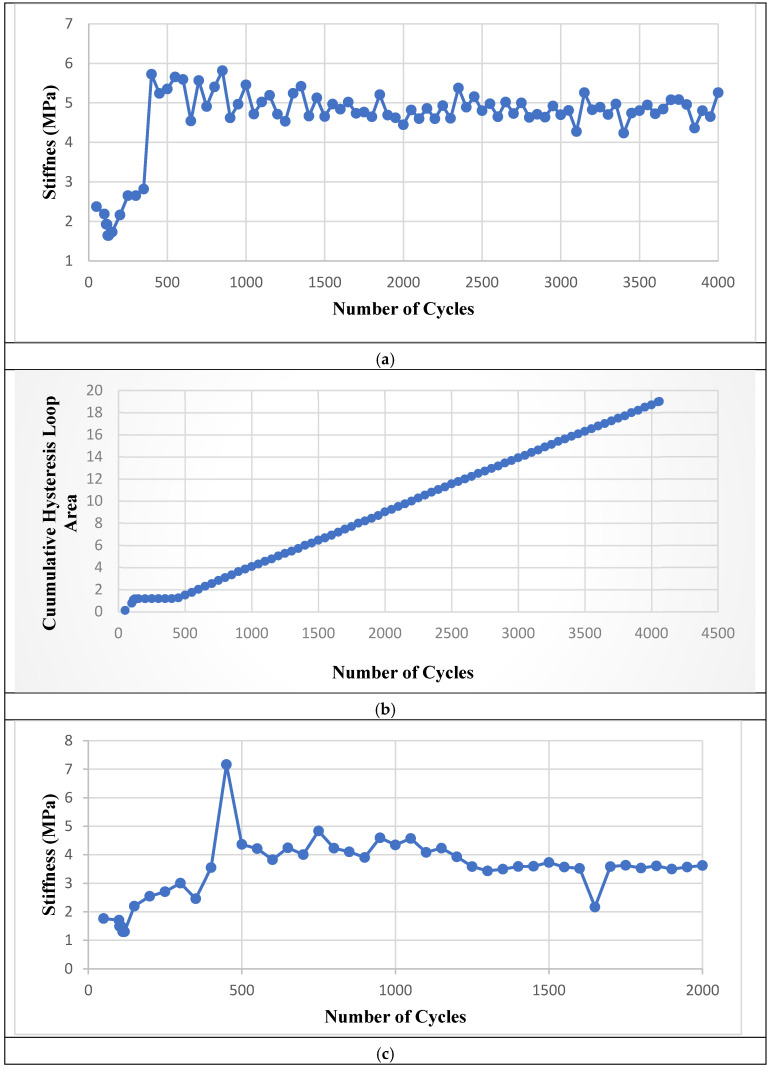

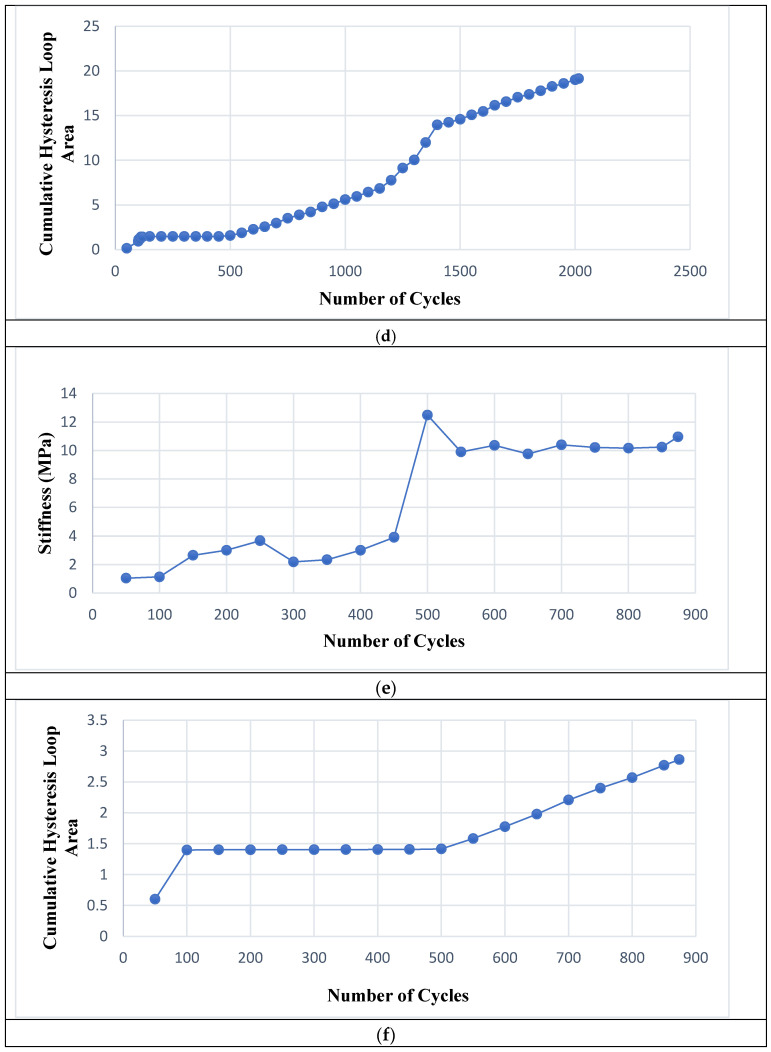
Fatigue analysis of asphalt mixtures: Malakand (**a**,**b**), Swabi (**c**,**d**), and Besai (**e**,**f**).

Based on the results for all three asphalt samples which are Malakand, Swabi, and Besai, they show the varying characteristics in performance in terms of stiffness and also cumulative hysteresis loop areas from four-point beam fatigue test. The Malakand sample showed excellent fatigue behavior in which the stiffness reaches quickly to stabilize around 5–6 MPa after going up and holding that way for nearly 4000 cycles. Cumulative hysteresis loop areas show a steady increase linearly instead of pace-wisely which gives that energy dissipation and the material integrity even under repeated loading depicted in Figure 15a,b. In the contrary, the Swabi sample shows a fairly average performance, with a maximum initial stiffness of about 7 MPa and gradually falls down with settling to a value of nearly 3.5 MPa, while at a faster accumulation of the hysteresis loop area after 1000 cycles shows a progressive damage due to fatigue, as depicted in Figure 15c,d. Hence, the Besai sample, while exceeding the threshold of more than 10 MPa, may give the upper limit after 500 cycles, but the premature failure occurs around the 900-cycle mark. The cumulative hysteresis loop area is significantly low, suggesting early failure and an inability to bear cyclic load, as shown in Figure 15e,f. Statistically, the Malakand sample endures the highest number of cycles with moderate stiffness and controlled energy loss, making it the most durable. The Swabi sample performs moderately well but shows signs of fatigue earlier, while the Besai sample exhibits poor fatigue resistance due to early failure despite its high stiffness. Therefore, the Malakand sample is classified as excellent, Swabi sample as average, and Besai sample as poor in terms of fatigue performance.

### 3.8. Performance Interpretation

Based on the macroanalysis, Margalla aggregates show the best overall performance, with the lowest abrasion (21%), flakiness (4.2%), elongation (4.6%), and water absorption (0.38%), as well as the highest fractured faces (99.3%). These values indicate high strength, durability, and good compaction ideal for surface and base layers of highways. Malakand and Kohat aggregates also meet all standard limits, though with slightly higher values. They are suitable for base or sub-base layers in medium-traffic roads. Swabi aggregates are within limits but show higher flakiness and elongation, which may affect compaction. They may be used in subbase or less critical areas. Besai aggregates perform the weakest, especially in abrasion (33.7%) and particle shape indices. Though still within limits, they are not recommended for surface layers and should be restricted to low-stress applications.

By studying the findings of microanalysis, the SEM analysis revealed that Margalla and Malakand aggregates exhibit angular and rough-textured surfaces with prominent fractured faces, which enhance mechanical interlocking with the asphalt binder beneficial for skid and rutting resistance. FTIR spectra confirmed the presence of silicates and carbonates, with strong Si-O and CO_3_ peaks across all samples, indicating typical mineral constituents of construction aggregates. EDS results supported this, showing high calcium content in Margalla and Malakand (indicative of limestone), while Kohat and Swabi showed increased silica and aluminum, suggesting a more silicate-rich composition. Overall, Margalla and Malakand displayed more favorable microstructural and mineralogical characteristics for highway applications, while Besai showed weaker surface morphology and composition, making it less ideal without modification.

Furthermore, by looking at the finding of mechanical testing, Margalla aggregates showed the best mechanical performance with the highest ITS, TSR, and lowest rutting, indicating superior strength, moisture resistance, and deformation resistance. Malakand and Kohat performed moderately well and are suitable for medium-load roads. Swabi and Besai had lower strength and higher rutting, making them less suitable for critical pavement layers. However, by designing aggregate gradation and bitumen content, these aggregates might be acceptable in high load applications as well. The reason is these aggregates showed promising results during the macroanalysis phase.

## 4. Conclusions

The current study evaluated the aggregates sourced from five different quarries of Pakistan to assess their and quality and suitability for pavement application. Extensive laboratory testing was conducted to evaluate the properties of these aggregates on macro-level, micro-level and performance testing in the lab. The aggregates from different sources were evaluated in reference to the aggregates sourced from Margalla Hills quarry. As a result of the extensive laboratory testing, the following conclusions were drawn:

1. Principal Component Analysis (PCA) showed that the first principal component (PC1) explains 95.96% of the total variance, indicating that the overall differences in aggregate quality among quarries can be effectively captured by a single composite indicator. Quarries like Margalla and Malakand scored highest on PC1, reflecting better physical and mechanical properties, while Besai and Swabi scored lowest, indicating inferior quality. This confirms that PCA can be a reliable tool for ranking aggregate sources based on combined performance attributes.
2. The Malakand and Kohat aggregates meet key performance standards and offer technically viable alternatives to Margalla. Utilizing these local sources from Khyber Pakhtunkhwa can reduce long-distance hauling, thereby lowering transportation costs, reducing carbon footprints, and minimizing project delays. This approach promotes sustainable construction practices by preserving depleted resources and encouraging the efficient use of regional materials.
3. SEM analysis revealed angular and rough-textured surfaces in both Margalla and Malakand samples, which enhance mechanical interlock and improve rutting resistance.
4. Based on EDS results, Margalla and Malakand aggregates, with high calcium content, exhibit superior physical and mechanical properties—high hardness, low impact value, low water absorption, and low porosity. Kohat and Swabi, due to mixed silicate and aluminosilicate content, show moderate hardness, higher impact values, and increased water absorption. Besai, with porous structure and fewer stabilizing elements, has the lowest hardness, highest water absorption, and poor resistance to impact, indicating inferior mechanical performance.
5. FTIR analysis identified distinct silicate and carbonate peaks in all samples, with stronger Si–O and CO_3_ bands observed in Margalla, followed closely by Malakand. While Kohat aggregates showed slightly weaker signals, the spectral characteristics remained consistent with those typically suitable for use in base and sub-base layers.
6. Balanced calcium content enhances adhesion and environmental resistance in Margalla and Malakand aggregates. In contrast, the higher reactive silica content in Swabi, Kohat, and Besai aggregates could affect binder compatibility and moisture susceptibility.
7. The Margalla aggregates exhibited superior mechanical strength, with the highest ITS and TSR values, the lowest rutting depth, and highest dynamic modulus, indicating excellent moisture resistance and deformation control. Malakand and Kohat aggregates also showed promising results that suggest their potential as partial alternatives.
8. The Malakand sample showed the best fatigue performance, maintaining stable stiffness (~5 MPa) for over 4000 cycles with a gradual hysteresis area rise (~19 units). The Swabi sample was moderate, with stiffness dropping from 7.2 to 3.5 MPa by 2000 cycles and a sharp hysteresis increase after 1200 cycles (~19 units). The Besai sample was poor, reaching peak stiffness (~12.5 MPa) early but failed by 900 cycles, with hysteresis area rising sharply after 500 cycles (~3 units).
9. Based on this multi-level evaluation, Malakand and Kohat aggregates may be considered as viable substitutes for Margalla aggregates in secondary and medium-load road applications, though Margalla remains the preferred choice for heavily trafficked pavements and high-stress environments.

Based on the current study, it was found that the aggregates from Margalla Hills quarry are the best performing aggregates. However, the aggregates from Malakand and Kohat also showed promising results for moderate traffic applications. On the other hand, Swabi and Besai aggregates particularly were lagging down in various aspects which makes them less suitable for pavement applications. However, it is recommended to evaluate the aggregates from these sources, mixed with Margalla, Malakand, or Kohat quarry in various proportions to see their combined performance. Adding more chemically stable and aggregates resistant to abrasion to the aggregates sourced from Swabi and Besai quarry may make them suitable for pavement applications.

## Figures and Tables

**Table 1:** Bitumen characterization.

Property	Value	Standard Deviation (±)	Limits	Ref.
Penetration (25 °C, 1/10th of mm)	62.4	0.56	60–70	[22]
Softening point (°C)	49.7	0.36	49–56	[23]
Ductility (cm)	103	2.00	>100	[24]
Flash and fire point (°C)	271, 288	2.00	>230, >250	[25]
Viscosity at 135 °C (Pa.s)	0.624	2.00	0.280–0.600	[26]
Viscosity at 165 °C (Pa.s)	0.173	0.006	0.15–0.2	[26]

**Table 2:** Asphalt mixtures’ volumetric properties.

Aggregate Source	OBC (%)	Bulk Density (g/cm^3^)	Marshall Stability (kN)	VMA (%)	VFA (%)	AV (%)	Dust-to-Binder Ratio
Margalla	4.20	2.46	14.8	15.5	76.5	3.65	1.19
Malakand	4.43	2.44	13.9	16.2	74.0	4.22	1.13
Kohat	4.87	2.41	12.8	16.8	70.3	5.00	1.03
Swabi	5.33	2.38	11.6	17.1	68.5	5.40	0.94
Besai	5.67	2.35	10.8	17.8	65.2	6.20	0.88

**Table 3:** Marshall design limits.

Parameter	Requirement
Marshall Stability	≥8.0 kN
Air Voids (AV)	3–5%
VMA	≥14%
VFA	65–75%
Flow	2–4 mm

**Table 4:** Consensus properties of aggregates.

Quarry	Margalla	Malakand	Kohat	Swabi	Besai	Standard	Recommended
Abrasion Value (%)	21	25.7	28.6	29.3	33.7	ASTM C131 [49]	≤30 (base) ≤35 (sub-base)
Flakiness Index (%)	4.2	7.6	8.7	12.8	14.4	BS 812-105 [50]	≤25
Elongation Index (%)	4.6	8.7	9.2	12.4	14.3	BS 812-105 [50]	≤30
Loss in Impact Value (%)	5.7	7.3	8.1	8.3	9.4	BS 812- 3 [51]	≤30 (wearing course) ≤40 (base)
Degree of Unsoundness (%)	5.2	8.1	9.7	9.8	11.2	ASTM C88 [52]	≤18
Fractured Faces (%)	99.3	98.6	98.1	97.4	95.2	ASTM D582 [53]	≥90
Water Absorption (%)	0.38	0.52	0.76	0.79	0.94	ASTM C127 [54]	≤2.0
Specific Gravity	2.89	2.81	2.7	2.68	2.57	ASTM C127 [54]	2.5–3.0

**Table 5:** PCA scores and ranking of aggregate sources.

Aggregate Source	PC1 Score	PC2 Score	PCA Rank
Margalla	4.284	−0.398	1
Malakand	1.481	0.132	2
Kohat	−0.336	0.706	3
Swabi	−1.491	0.007	4
Besai	−3.938	−0.446	5

**Table 6:** Variance explained by principal components.

Principal Component	Variance Explained (%)
PC1	95.96
PC2	2.18
PC3	1.33
PC4	0.53
PC5	~0.00

**Table 7:** Normalized score for each quarry.

Quarry	Abrasion	Flakiness	Elongation	Impact Loss	Unsoundness	Fractured Faces	Water Abs.	Specific Gravity	Total Score
Margalla	1.00	1.00	1.00	1.00	1.00	1.00	1.00	1.00	8.00
Malakand	0.79	0.67	0.62	0.86	0.52	0.83	0.75	0.75	5.79
Kohat	0.60	0.56	0.53	0.73	0.25	0.71	0.32	0.41	4.11
Swabi	0.56	0.16	0.20	0.70	0.23	0.54	0.27	0.34	3.00
Besai	0.00	0.00	0.00	0.00	0.00	0.00	0.00	0.00	0.00

**Table 8:** Aggregate’s performance interpretation based on PCA.

Quarry	Total Score	Rating	Performance Summary
Margalla	8.00	Excellent	Best in every category.
Malakand	5.79	Very Good	Strong runner-up, but lags in Flakiness/Elongation.
Kohat	4.11	Good	Mid-tier, weaknesses in Unsoundness/Water Absorption.
Swabi	3.00	Fair	Poor Flakiness/Elongation, decent Impact Loss.
Besai	0.00	Poor	Worst in all metrics.

## Data Availability

The raw data supporting the conclusions of this article will be made available by the authors on request.
